# Porcine Vaccines Contribute to Enhancing Health, Productivity, and Animal Welfare

**DOI:** 10.3390/vaccines14040333

**Published:** 2026-04-09

**Authors:** Zichen An, Shengwei Di, Tongqing An

**Affiliations:** 1 College of Animal Science and Technology, Northeast Agricultural University, Harbin 150030, China; 2 State Key Laboratory of Animal Disease Control and Prevention, Harbin Veterinary Research Institute, Chinese Academy of Agricultural Sciences, Harbin 150069, China

As a major source of animal protein, pork production is directly related to food safety. However, outbreaks and epidemics of infectious diseases can not only result in illness or the death of pigs, along with huge economic losses to the pig industry, but also seriously threaten its productive performance and animal welfare. Pigs can be natural hosts to a variety of pathogens, some of which cause zoonotic infectious diseases, such as Japanese encephalitis virus (JEV), hepatitis E virus (HEV) genotype 3, pseudorabies virus (PRV), and *Streptococcus suis* type 2 (SS2) [1,2,3]. The effective prevention and control of swine diseases can not only enhance the health of pigs but also reduce the transmission of many zoonotic infectious diseases from swine to humans.

Vaccination is the most economical and effective strategy for preventing and controlling infectious diseases in animals. In general, the evaluation of vaccine immunization effects mainly focuses on the induction of efficient immune responses, the reduction in disease incidence, and the alleviation of disease severity. The effects of vaccine immunization can go beyond the “disease prevention” function and extend to promoting growth, improving reproduction, raising animal welfare, and decreasing the risk of antimicrobial resistance. Under the immunological protection provided by vaccination, excellent breeding performance can be achieved. In China, although the scale of pig farming ranks among the top in the world, the number of market pigs provided by each sow per year (MSY) still lags behind that of leading pig-producing countries, such as Denmark [4]. Disease is one of the most important factors affecting the production performance of pigs. In the Special Issue entitled ‘Porcine Vaccines: Enhancing Health, Productivity, and Welfare’ in the journal *Vaccines*, we draw attention to the development and evaluation vaccines against pig diseases in order to share advanced outcomes of porcine vaccines.

African swine fever virus (ASFV) is currently the biggest threat to the global swine industry. In a recent study, the MGF505-2R protein of ASFV was identified as an inhibitor of the cGAS/STING pathway and found to be involved in controlling IFN-β production [5]. Subsequently, a recombinant ASFV lacking the MGF505-2R gene (Arm/07-ΔMGF505-2R) was generated, and this recombinant virus was remarkably attenuated in piglets. More importantly, immunization with Arm/07-ΔMGF505-2R proved to be partially protective against the wild-type virulent ASFV strain [5]. With the gradual discovery of virulence-related genes in ASFV, the development of live attenuated vaccines against ASFV based on virulence-related gene deletions has further advanced. Inactivated viral vaccines offer excellent safety; however, inactivated vaccines have been confirmed to be ineffective against ASFV. The immunological protective capability of inactivated vaccines varies among different viruses. Recently, the immunogenicity of an inactivated Senecavirus A (SVA) vaccine was evaluated using a representative contemporary Brazilian SVA strain in mice. In that study, the inactivated SVA vaccine induced high levels of humoral and cellular responses in a murine model [6]. The findings may guide a further pre-clinical study into the effects of the inactivated SVA vaccine on pigs.

Enterotoxigenic *Escherichia coli* (ETEC) is a pathogenic strain of *Escherichia coli* that can cause diarrhea in humans and various animals [7]. ETEC primarily affects young animals, resulting in diarrhea and dehydration in piglets [7,8]. For a long time, the main approach to ETEC control relied on antibiotics; however, as a result of their use, the number of drug-resistant bacteria has increased, posing a challenge to the treatment of bacterial diseases in humans. Recently, an oral vaccine for ETEC was generated by presenting the LTA subunit, the LTB subunit of heat-labile enterotoxin, and the FaeG of F4+ ETEC on the surface of recombinant *Lactococcus lactis* (LAB) [7]. The vaccine was found to provide effective protection against F4+ ETEC infection in mice and piglets. More importantly, sow immunization during late pregnancy generated significantly elevated antibodies in colostrum, which protected piglets against F4+ ETEC infection. The recombinant LAB live vector oral vaccine described in this study provided a promising vaccination strategy for the prevention and control of ETEC-induced diarrhea in piglets [7]. Vaccines for ETEC are currently an interesting research hotspot. In another study, the Enteroporc Coli AC^®^ vaccine, containing fimbrial antigens F4ab, F4ac, F5, and F6 of ETEC and alpha and beta toxoids of *Clostridium perfringens* type C and type A (including beta2 toxoid), was proven to be effective under field conditions on a pig farm [8].

As subunit vaccines are composed of only one or a few non-infectious proteins, virulence reversion is avoided compared to live attenuated vaccines. The efficacy of subunit vaccines is highly dependent on their antigen components. In a recent study, the formulation of a subunit vaccine candidate against *Lawsonia intracellularis* was readjusted, with a ratio of three chimeric antigens set at 1:1:1 [9]. As a result, the new subunit vaccine induced a significant immune response in inoculated pigs that persisted until the end of the experiment. Animals showed consistent weight gain and normal clinical signs during immunization assays. The results indicate that the relative proportion of subunit vaccine components represents a critical parameter in vaccine optimization, and an optimized formulation ratio is expected to enhance immunogenicity.

The combined usage of vaccination and iron treatment is an efficient approach in clinical practice. Sperling et al. [10] simultaneously used iron/anticoccidial treatment and vaccination against Oedema disease (OD) to evaluate any effect on efficacy. As a result, under the conditions of the study, the efficacy of OD vaccination and iron/anticoccidial treatment was not affected by simultaneous use. This approach can reduce the number of animal immobilizations and injections, thereby alleviating the pain experienced by the animals. This is also one of the reasons why there is a high demand for multivalent vaccines in clinical practice.

Reproductive diseases are the main cause of low production efficiency in sows; moreover, various viral diseases are core factors causing reproductive disorders, including porcine reproductive and respiratory syndrome virus (PRRSV), porcine parvovirus 1 (PPV1), classical swine fever virus (CSFV), PRV, and JEV. Effective vaccination against these pathogenic microorganisms will safeguard sow reproductive performance and enhance pre-weaning piglet survival. Vaccination can also significantly contribute to animal welfare. Animal diseases induce substantial physiological distress—including pyrexia, nociception, dyspnea, and diarrhea—whereas effective vaccination prevents disease onset and thereby eliminates associated suffering. Moreover, vaccination will mitigate reliance on therapeutic antibiotics and other pharmacological interventions, reducing both iatrogenic stress and procedural discomfort.

Multivalent vaccines are becoming important trend in porcine vaccine research and development. For instance, concurrent administration of vaccines against *Mycoplasma hyopneumoniae*, porcine circovirus type 2 (PCV2), and CSFV has been empirically validated to yield no significant antigenic interference, enabling protocol simplification, and thereby reducing labor intensity for immunization personnel and minimizing stress in pigs. In the future, development efforts are expected to prioritize multivalent vaccines associated with the same clinical syndrome, such as swine-reproduction-related diseases or respiratory-related diseases. This is because a rationally designed multivalent vaccine will significantly enhance the integrated prevention and control of similar diseases.

Needle-free intradermal delivery (NFID) represents a new approach in swine vaccination. NFID utilizes high-pressure jet technology to deliver the vaccine into the immunologically active dermal layer. This approach markedly attenuates pain and stress responses, diminishes cross-infection potential, enhances operator safety and procedural efficiency, and allows for dose-sparing effects. Currently, this immunization technology is being increasingly adopted in large-scale pig farms.

In summary, the strategic development of swine vaccines will increasingly prioritize the integrated optimization of safety, efficacy, animal welfare, and cost-effectiveness. Vaccination, biosecurity, nutrition, and genetic selection will collectively constitute the four foundations of modern swine production. This framework is essential for advancing the global pork industry toward enhanced health, higher productivity, and better animal welfare.
